# Forensic chemical analysis of hydrogen gas explosions in sprinkler pipes

**DOI:** 10.1038/s41598-023-41973-x

**Published:** 2023-09-15

**Authors:** Songhie An, Sungwan Park

**Affiliations:** 1 Department of Forensic Toxicology and Chemistry, National Forensic Service Seoul Institute, 139 Jiyang-Ro, Yangcheon-Gu, Seoul, 08036 Republic of Korea; 2 Department of Engineering, National Forensic Service Seoul Institute, 139 Jiyang-Ro, Yangcheon-Gu Seoul, 08036 Republic of Korea

**Keywords:** Chemistry, Engineering

## Abstract

Most sprinkler systems in Korea use galvanized steel pipes to extinguish fires. Typically, sprinkler pipes are galvanized with zinc to protect them from corrosion. Although such installations are widely perceived to be safe and hazard-free, the corrosion of zinc within water-filled sprinkler pipes leads to the formation of corrosive products, including hydrogen gas. As hydrogen gas evolves, the pressure inside the closed sprinkler system increases, which in turn increases the risk of fire explosion. In this study, we evaluated several factors that contributed to the formation of hydrogen gas and analyzed the chemical principle of this reaction. Based on the results, we propose certain safety measures that can aid in preventing accidents caused by hydrogen gas. The study findings can form the basis for identifying a safe operating method that can prevent fires and explosions in sprinkler systems.

## Introduction

Steel pipes galvanized with zinc are primarily used in sprinkler systems to prevent corrosion and are installed in most buildings as a safe standard fire extinguishing system. As outlined by Zalosh^1^ and Park et al.^2^, several explosions caused by hydrogen gas formation in sprinkler systems have been reported in Scandinavia^3,4^, France^1^, and Korea (Table 1). Although the possibility of hydrogen gas formation due to corrosion in galvanized sprinkler pipes was raised by Yamate et al.^5,6^ over a decade ago, academic literature on hydrogen explosion in sprinkler systems is still limited.

**Table 1:** Summary of fire and explosion incidents caused by hydrogen gas in sprinkler systems.

Item	Year	Description	Country
1	2014	A sprinkler contractor was burned by a hydrogen flame	Denmark
2	2014	A sprinkler contractor was burned	Denmark
3	2016	An explosion occurred in a sprinkler fire pump house	France
4	2020	An explosion caused burn injuries to the contractor	Finland
5	2020	An explosion occurred in a water-collecting tank	Republic of Korea
6	2021	An explosion occurred in a water-collecting tank	Republic of Korea

The National Forensic Service in Seoul investigated two of such explosions in the past 2 years^2^, wherein the formation of flammable gas within the pipes contributed to these accidents. Forensic examinations ruled out criminal activity. This report confirmed the emission of hydrogen gas from sprinkler pipes at the explosion site. In this case, high levels of hydrogen gas were emitted from the pipes into the water-collecting tank while draining the pipes. Subsequently, the hydrogen gas combined with the ignition source and caused the explosion. Typically, the formation of hydrogen gas can be attributed to the simple redox reaction of a metal with water, where the metal forms an oxide and releases gaseous hydrogen. In this study, we performed a proof-of-concept experiment, wherein zinc disks were immersed in regular tap water and deionized water (DW) in a vial to observe the chemical changes occurring on the metal surface, within the water, and in the headspace (HS). Additionally, water and white precipitates collected from the incident sites were analyzed to verify our hypothesis. Several groups in Norway^3^ and the US^7^ have provided guidelines and safety precautions for the aforementioned issues. These guidelines were partially considered in this study in addition to analyzing the safety measures that should be applied to sprinkler systems to prevent further accidents caused by hydrogen explosions.

The objective of this study was to complement our previous analysis on hydrogen gas explosions in sprinkler systems. We analyzed water samples collected from the incident site along with the parameters within the sprinkler system that contributed to the formation of hydrogen gas. Furthermore, the chemical principle of this reaction was investigated, based on which safety guidelines are recommended to prevent similar accidents in the future.

## Materials and methods

### Water flow in sprinkler systems at the incident site

**Figure 1:**
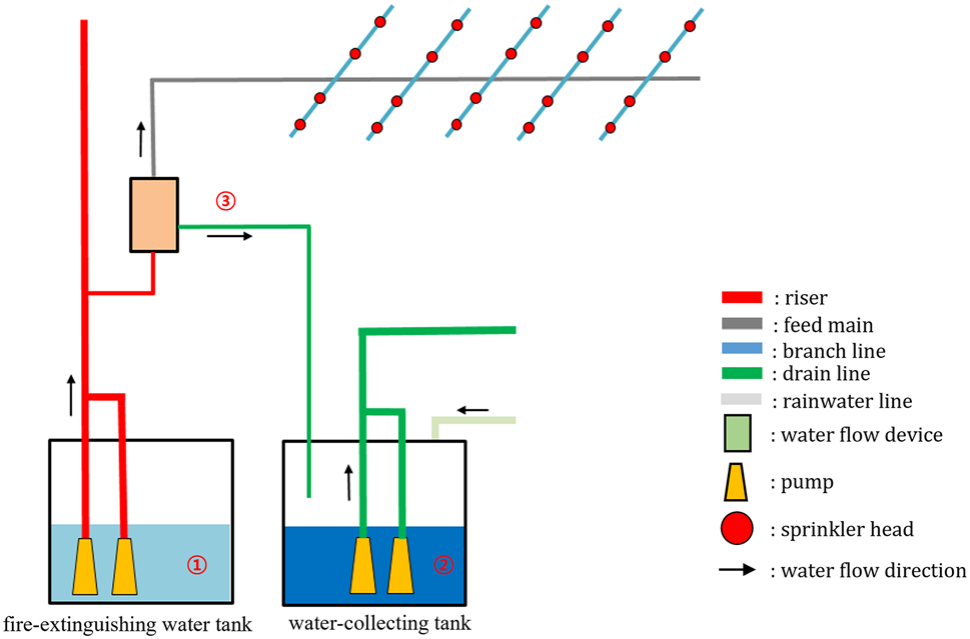
Schematic of the water flow in sprinkler systems.

Figure 1 depicts a schematic of the sprinkler system, which automatically detects and extinguishes the fire by spraying water. When water is sprayed through the sprinkler head, the pressure difference between the riser and feed main pipe opens the clapper in the alarm valve. The pump detects the pressure drop and provides a continuous water supply. When disassembling or installing a device for sprinkler system maintenance, the water in the pipe is moved to the water-collecting tank through the drain line. The water level in this water-collecting tank is controlled using the pump and water level control sensor.

### Sample collection at the incident site

At the incident site (Fig. 1), samples of tap water (①), fire-extinguishing water, water from inside the water-collecting tank (②), and water from inside the sprinkler pipe on the 24th floor (③) were collected in addition to the precipitates formed within the sprinkler pipes. All samples were collected by following appropriate safety procedures with permission from the management office.

### Hydrogen gas formation

To reproduce hydrogen gas formation within the sprinkler pipes, zinc disks with a diameter of 1 cm were fabricated (Jisi industry) and immersed in 10 ml of tap water, DW, and seven different brands of alkaline drinking water (samples 1–7) in a glass vial for 14 days (Table 2). HS samples were collected on days 0, 2, 7, and 14; the analysis for hydrogen gas was immediately performed using a gas chromatography thermal conductivity detector (G1530A, Agilent Technologies) equipped with a molecular sieve column (5 Å; packed 60/80, 10 ft, 2.0 mm).

**Table 2:** Changes in the pH of water samples.

Samples	pH pre-immersion	pH post-immersion	pH difference	Concentration of formed hydrogen gas (ppm)		
				Day 0	Day 7	Day 14
Tap water	7.71	11.00	3.28	0	1076	1399
DW	7.06	9.51	2.45	0	2098	2315
1	7.44	10.83	3.39			
2	8.72	10.25	1.53	0	675	1161
3	7.85	11.16	3.31			
4	7.87	9.17	1.31			
5	9.12	10.17	1.05	0	936	1153
6	8.19	11.23	3.05			
7	8.09	9.97	1.88			

The water samples analyzed include tap water, deionized water, and alkaline water samples 1–7. The results were obtained after 14 days of the samples being in contact with zinc disks. Hydrogen gas concentration (ppm) formed in the headspace of tap water, deionized water, sample 2 and 5 was also measured.

### Water analysis

The pH of each water sample was measured in triplicate using a waterproof handheld pH meter (IQ Scientific Instruments). The ionic composition of each water sample was determined by injecting 200 μL samples into an ion chromatograph (IC; DIONEX ICS-6000 DC, Thermo Scientific) equipped with a Dionex IonPac™ AS19 column (Thermo Scientific). Quantitative analysis was performed with Chromeleon based on calibration curves prepared with cation and anion standard solutions (Multi Component Cation Mix 1(IC-MCI-01-1), Multi Component Anion Mix 1(IC-MAN-01-1); AccuStandard). Data of water samples collected at the incident site (Fig. 1 indicated as , , and ) were obtained on the day of collection, whereas those of the water samples that had been in contact with zinc disks (as described in “Hydrogen gas formation”) were measured on day 0 (before the immersion of zinc disks) and day 14.

### Elemental analysis of zinc disks and precipitates

Zinc disks were analyzed using scanning electron microscopy-energy dispersive X-ray spectroscopy (SEM–EDS; COXEM (NanoStation), Oxford (AztechOne)) before and after immersion in each water sample. White precipitates that accumulated at the bottom of each vial with zinc disks were collected after 14 days. Precipitates accumulated on the sprinkler pipe wall at the incident site were collected in glass vials. All samples were air-dried at 25 °C for 2 h and analyzed using a Raman spectroscopy (DXR3 Raman Microscope; Thermo Scientific).

## Results

### Hydrogen gas formation and water analysis

**Figure 2:**
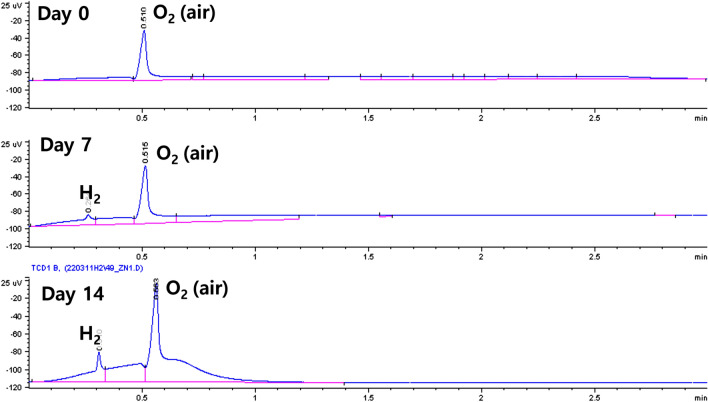
Hydrogen gas formation in the headspace of water containing zinc disks.

Hydrogen gas was detected in the HS samples of vials containing zinc disks immersed in water (Fig. 2).

The hydrogen gas concentration reached a maximum value and plateaued on day 7 (Table 2); at this point, white precipitates covered the surfaces of zinc disks. The pH of each water sample increased over time by approximately 1 to a maximum value of 3.39 when in contact with zinc (Table 2).

The degree of change in pH increased because the pH values of the water samples before the immersion of zinc disks were low. In other words, the water samples were relatively richer in proton concentration, except in the case of drinking water (sample 4). No significant difference was observed in the degree of pH change between tap water and DW. However, compared to the most alkaline water (sample 5), more hydrogen gas was formed as the pH of the water was initially lower. Additionally, water samples from the incident site indicated that the municipal water (fire-extinguishing water) had a pH of 8.0 before entering the sprinkler pipes. Conversely, the pH of the water collected from the 24th floor was approximately 10 and that of the water drained from the sprinkler pipes into the water-collecting tank was approximately 9.7. This increase in pH indicated a decrease in the proton (${\text{H}}^{+}$) concentration, which was caused by the formation of hydrogen gas (${\text{H}}_{2}$) when water was inside the sprinkler pipes and in contact with the zinc coating. The slight decrease in the pH of the water samples from the water-collecting tank in comparison with that of the samples from the 24th floor could be attributed to the external exhaust gas contamination and inflow of external materials through the rainwater line owing to the structure of the water-collecting tank and its location in the garage; however, this was not verified in the study.

**Figure 3:**
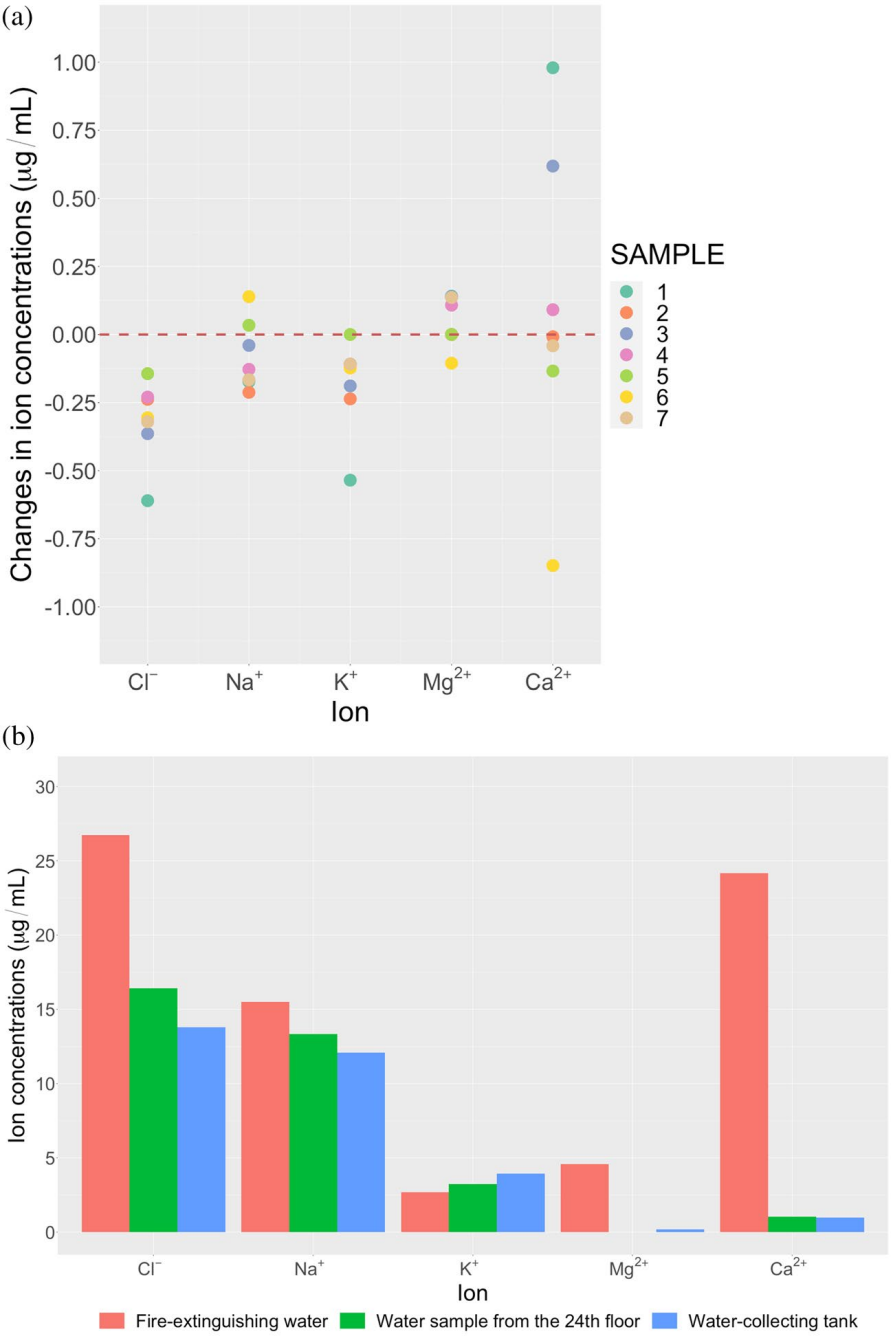
Changes in ion concentrations (μg/mL) after immersion of zinc disks in (**a**) alkaline water samples 1–7 and (**b**) water samples collected from three different locations at the incident site.

No significant change was observed in the ionic concentration and composition of alkaline drinking water before and after immersion of zinc disks when analyzed using an IC (Table 3, Fig. 3). The results also show that pH plays a more dominant role in hydrogen gas formation as alkaline drinking water sample 2 and 5, both similar in pH but different in ionic composition, produced a similar amount of hydrogen gas (Table 2). Conversely, zinc oxidation and hydrogen gas formation could not be associated with a significant change in ionic composition in each alkaline drinking water.

**Table 3:** Changes in ion concentration (μg/mL) in water before and after zinc disk immersion.

Sample		Ion concentration (μg/mL)				
		[Cl^−^]	[Na^+^]	[K^+^]	[Mg^2+^]	[Ca^2+^]
1	Pre-immersion	0.6103	4.8943	0.9162	1.4423	8.0314
	Post-immersion	0	4.7216	0.3812	1.5831	9.0109
2	Pre-immersion	0.2382	2.1996	0.2364	–	0.7287
	Post-immersion	–	1.9872	–	–	0.7204
3	Pre-immersion	1.7473	3.3538	1.0154	1.2759	7.4255
	Post-immersion	1.3832	3.3138	0.8265	1.4144	8.0439
4	Pre-immersion	4.8949	18.8677	1.5073	13.54	14.4032
	Post-immersion	4.665	18.7398	1.3981	13.6478	14.4942
5	Pre-immersion	0.1438	1.7737	–	–	0.8185
	Post-immersion	–	1.8077	–	–	0.6843
6	Pre-immersion	0.9324	12.805	1.1711	1.8888	7.8819
	Post-immersion	0.6266	12.9439	1.048	1.7833	7.0334
7	Pre-immersion	2.7924	6.2958	1.3224	4.5046	11.08
	Post-immersion	2.472	6.1296	1.2139	4.6406	11.0387
Fire-extinguishing water (tap water)		26.7175	15.4889	2.6946	4.5819	24.1659
24th floor		16.4271	13.3267	3.2273	–	1.0498
Water-collecting tank		13.7998	12.097	3.9388	0.1667	0.9773

Cells filled with ‘–’, and no numbers, indicated that the measured concentration was below the limit of detection.

Table 3 also shows that fire-extinguishing water (tap water), prior to entering the sprinkler system, is significantly higher in ${\text{Cl}}^{-}$, ${\text{Mg}}^{2+}$, and ${\text{Ca}}^{2+}$ ion concentrations compared to alkaline drinking water. These levels decrease as the water is kept within the sprinkler system (24th floor) and eventually is released into the water-collecting tank.

### Oxidation of zinc

**Figure 4:**
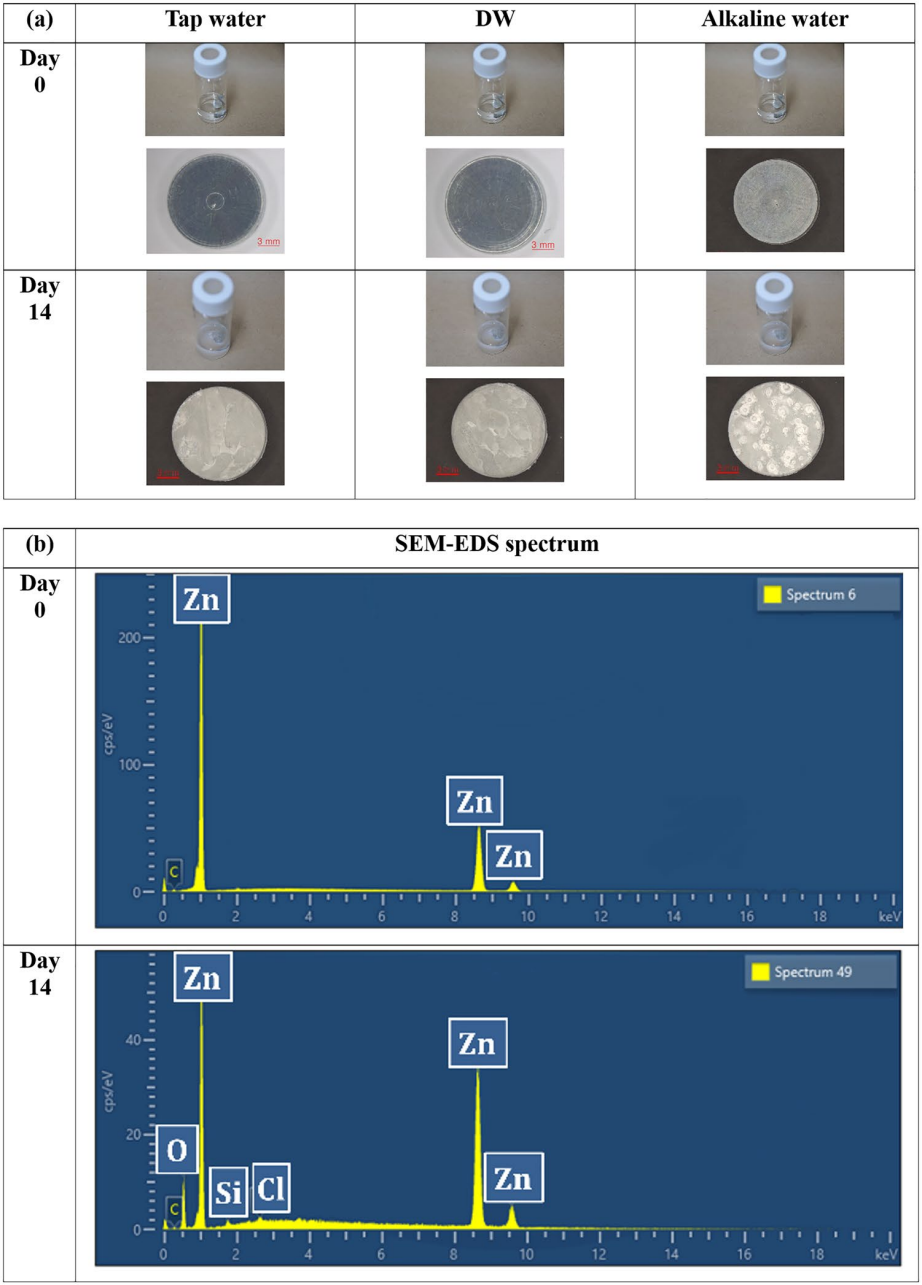
Images of (**a**) white precipitate formed during the zinc disk experiment in tap water, deionized water (DW), and alkaline water, and (**b**) representative scanning electron microscopy-energy dispersive X-ray spectroscopy (SEM–EDS) results on days 0 and 14.

SEM–EDS analysis of the zinc disks indicated that after 14 days of immersion in water, the surface composition changed from zinc to an oxidated form of zinc (Fig. 4). The initial atomic composition of the disks was mainly zinc, but over time, white precipitates composed of zinc and oxygen formed on the surface. Traces of silica (Si) and chlorine (Cl) were also detected.

**Figure 5:**
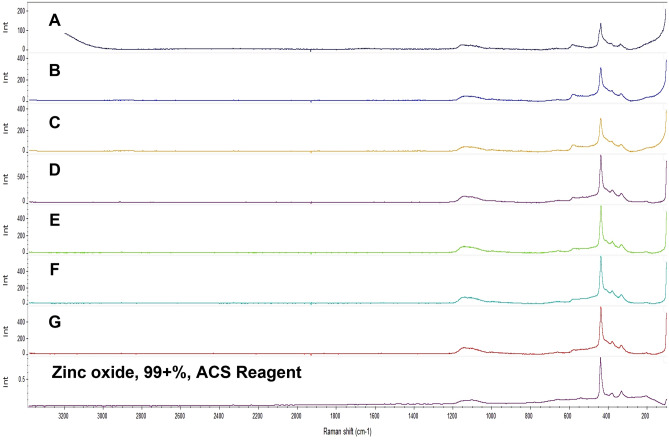
Raman spectroscopy results of white precipitates collected from the incident site (A) and pulverized precipitates that accumulated at the bottom of the vials with zinc disks and tap water (B, C), deionized water (DW) (D, E), alkaline water (F, G), and a standard zinc oxide. Characteristic peaks of zinc oxide are visible at around 437, 380, 330, and 97 cm^−1^.

This result was in line with the previously reported analytical results of white precipitates collected from the incident site^2^ and other related studies^8^. The Raman spectroscopy results verified that the white pulverized precipitates accumulated at the bottom of the vials and those collected from the incident site were also an oxidized form of zinc (Fig. 5).

## Discussion

In this study, we obtained experimental evidence from lab-controlled samples and samples collected from an incident site, where the pH of the water in contact with zinc increased over time, producing hydrogen gas and oxidized zinc. As water samples exhibited higher hydrogen ion concentrations (lower pH), the pH change was greater when water was in contact with zinc (except in the case of one alkaline drinking water sample). Consequently, more hydrogen gas was produced than that observed in regular tap water with a pH of 7.7 and DW over a period of 14 days.

The following equations explain the aqueous system (water) supplying [${\text{OH}}^{-}$] to the metal to form the white precipitate, zinc hydroxide (Zn(OH)_2_), and releasing hydrogen (${\text{H}}_{2}$) gas into the air.

1 $${\text{2H}}_{{2}} {\text{O}} \to {\text{2H}}^{ + } + {\text{ 2OH}}^{ - }$$

2 $${\text{Zn}} \to {\text{Zn}}^{{{2} + }} + {\text{ 2e}}^{ - }$$

3 $${\text{2H}}^{ + } + {\text{ 2e}}^{ - } \to {\text{ 2H}}_{{2}}$$

4 $${\text{Zn}}^{{{2} + }} + {\text{ 2OH}}^{ - } \to {\text{ Zn}}\left( {{\text{OH}}} \right)_{{2}}$$

Bernard et al.^9^. summarized that Zn(OH)_2_ in Eq. (4) can further undergo chemical reactions in the presence of chloride to form different forms of ZnO which is stable at a pH higher than 8.5 and low chloride concentration. This is in line with our Raman results, in which all water samples were above a pH 9 after 14 days of zinc disk immersion.

Previous studies have reported an abundance of ions within water, facilitating a higher electron potential that accelerated zinc oxidation and hydrogen gas formation^8,10–12^. Although this trend could not be verified using the laboratory-scale experiments performed in this study, our analysis confirmed that water samples collected from different areas at the incident site exhibited different ionic concentrations. This shows that the water inside the sprinkler pipes is indeed undergoing chemical reactions that involve the exchange of electrons, possibly facilitating hydrogen gas formation, as it travels along the pipes.

As mentioned in our previous study^2^, the formation of hydrogen gas is generally considered to be a slow reaction occurring in an open system where gas is easily diffused. Therefore, in many countries where galvanized carbon steel pipes (coated with zinc) are used, the generation of hydrogen gas inside the pipes and the potential risk of explosion are not mentioned in the installation and operation regulations of sprinkler systems. Moreover, the chemical reactions within sprinkler pipes are typically perceived to be harmless or have minimal consequences. Thus, potential hazards were previously disregarded. However, this study verified that a build-up of the reaction product occurs within weeks, leading to dangerous incidents that can pose a threat to people. This implies that preventive actions are necessary along with the reformation of sprinkler systems and their standard requirements. Further research is needed to quantify hydrogen gas levels built from corroded sprinkler pipes and connect those results with levels of explosion risk as a function of time, properties of water, pipe dimensions, etc.

In the case of sprinkler systems that are planned to be installed in the future, hydrogen gas generation can be prevented by limiting the use of zinc-coated pipes; however, sprinkler systems with zinc-coated pipes that are currently installed present the risk of a hydrogen explosion. To prevent fires and explosions, regular checks, and removal of hydrogen from inside the pipes should be performed. Furthermore, installing a ventilation system that releases built-up gas within the sprinkler pipes into the open air would be useful. Regular monitoring of water pH and ionic concentrations can also alert workers and managers to exercise precautions when working on sprinkler pipes. Thus, our study findings can contribute to the safe operation of sprinkler systems by understanding the generation and progress of hydrogen gas in pipes well in advance.

## Data Availability

All data generated or analyzed during this study are included in this published article.
